# Simultaneous Liver-kidney Versus Liver Transplantation Categorized by Pretransplant eGFR: Impact of the 2017 OPTN Policy

**DOI:** 10.1097/TP.0000000000005776

**Published:** 2026-06-30

**Authors:** Yitian Fang, Sarah Bouari, Jacqueline van de Wetering, Caroline M. den Hoed, Ron W.F. de Bruin, Robert J. Porte, Wojciech G. Polak, Robert C. Minnee

**Affiliations:** 1 Department of Surgery, Division of HPB and Transplant Surgery, Erasmus MC University Transplant Institute, Rotterdam, the Netherlands.; 2 Department of Internal Medicine, Section of Nephrology and Transplantation, Erasmus MC University Transplant Institute, Rotterdam, the Netherlands.; 3 Department of Gastroenterology and Hepatology, Erasmus MC University Transplant Institute, Rotterdam, the Netherlands.

## Abstract

**Background.:**

Simultaneous liver-kidney transplantation (SLKT) has increased substantially in the United States since adoption of the Model for End-stage Liver Disease-based allocation, now comprising 9%–10% of liver transplants. The survival benefit of SLKT compared with liver transplantation alone (LTA) remains uncertain.

**Methods.:**

Using the Organ Procurement and Transplant Network (OPTN)/United Network for Organ Sharing registry, we analyzed 63 407 adult liver transplants (57 780 LTA; 5627 SLKT) performed between 2010 and 2019. Patient survival was evaluated across pretransplant estimated glomerular filtration rate (eGFR) categories and according to transplant era before and after implementation of the 2017 OPTN policy.

**Results.:**

Among patients with pretransplant eGFR ≥30 mL/min/1.73 m^2^, SLKT conferred no survival benefits, and LTA recipients achieved comparable or better long-term outcomes. In contrast, SLKT significantly reduced mortality in patients with advanced renal dysfunction. For eGFR 15–29 mL/min/1.73 m^2^, SLKT reduced 1-, 3-, and 5-y mortality by 25%, 15%, and 13%, respectively. For eGFR <15 mL/min/1.73 m^2^, SLKT reduced 1-, 3-, 5-, and 10-y mortality by 32%, 24%, 22%, and 17%, respectively. Following implementation of the 2017 OPTN policy and “Safety Net” policies, SLKT procedures declined. The survival advantage of SLKT observed in the pre-policy era was no longer evident post-policy, with 1.4% of LTA recipients undergoing kidney after liver transplantation under “Safety Net” allocation priority.

**Conclusions.:**

SLKT is associated with survival benefit in patients with severe renal dysfunction (eGFR <30 mL/min/1.73 m^2^). Following implementation of the 2017 OPTN policy, this survival difference was no longer evident, and SLKT procedures were reduced without compromising survival. These findings support a stage-specific allocation strategy incorporating the “Safety Net” framework.

## Graphical abstract

**Figure:**
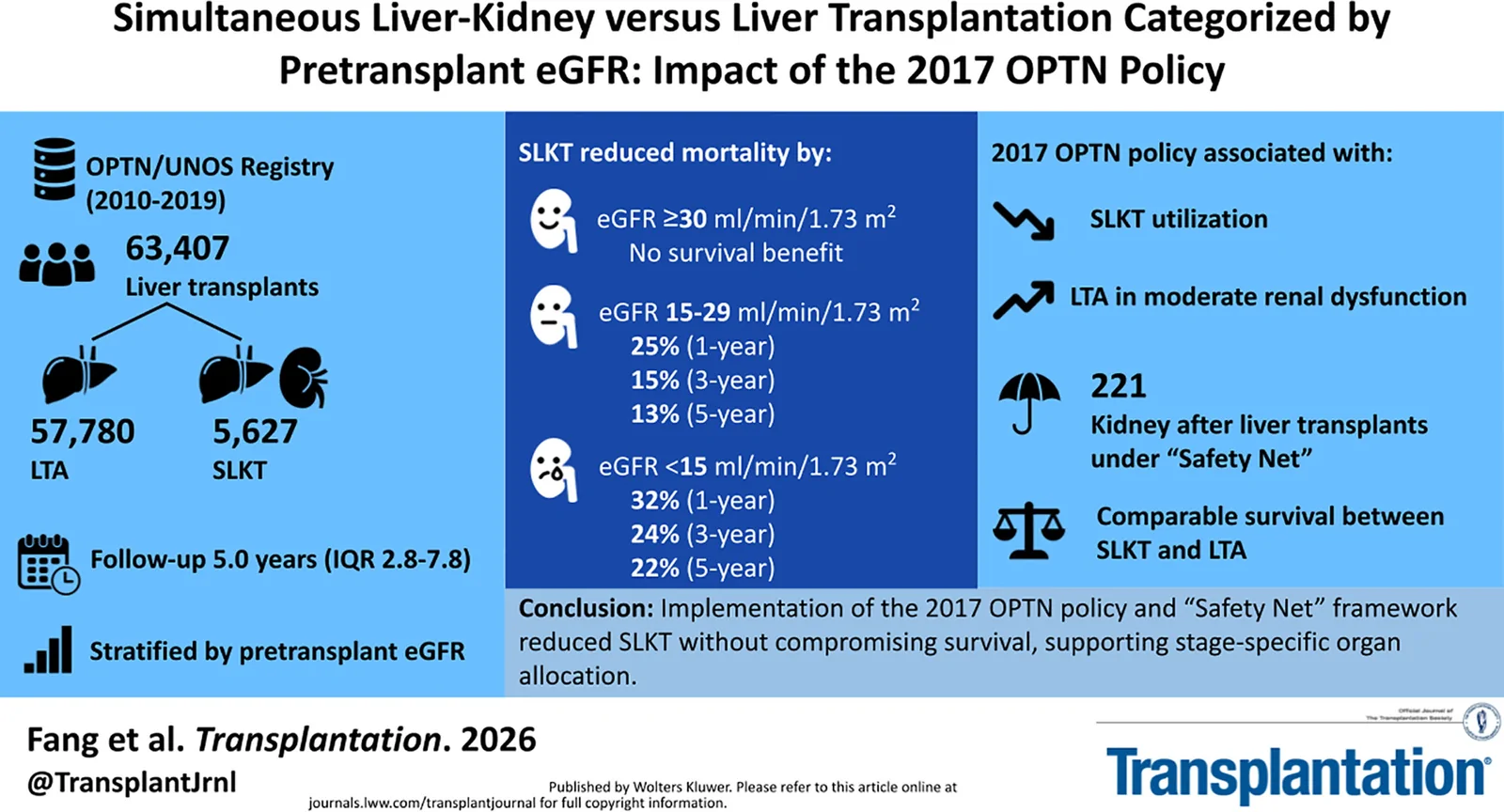

## INTRODUCTION

Simultaneous liver-kidney transplantation (SLKT) provides a life-saving option for patients with both end-stage liver disease (ESLD) and end-stage renal disease.^1^ One of the major comorbidities of ESLD is portal hypertension, which causes pooling of the portal blood in the splanchnic circulation. This reduces the effective circulating volume and increases the risk of renal dysfunction and acute kidney injury.^2^ Patients with ESLD and decompensated cirrhosis are further at risk of developing chronic kidney disease (CKD) because of hepatorenal syndrome, hypoperfusion, parenchymal renal disease, and drug-induced nephrotoxicity.^3,4^

SLKT has become the most common multiorgan transplantation in the United States, as classified by United Network for Organ Sharing (UNOS). It has been performed increasingly since the Organ Procurement and Transplant Network (OPTN) adopted the Model for End-stage Liver Disease (MELD) scoring system for organ allocation in 2002.^5,6^ Since impaired renal function contributes to higher MELD score, patients with concomitant renal injury are more likely to be prioritized for liver transplantation, thereby contributing to the rising frequency of SLKT procedures. In 1998, only 23 SLKTs were performed, accounting for 1.3% of all deceased-donor liver transplants. However, the total number of SLKTs has now reached 700 per year, making approximately 9%–10% of deceased liver transplants in the United States.^7,8^

Despite the steady increase, the limited supply of donor organs remains a challenge for both SLKT and single-organ transplants. Moreover, recent evidence suggests that SLKT does not fully mitigate the negative impact of pretransplant CKD on long-term survival, as systemic consequences of CKD, such as cardiovascular disease, metabolic complications, and frailty may be irreversible.^9^ These concerns prompted the OPTN to restrict eligibility for SLKT in 2017.^6,8,10^ Meanwhile, the new policy “Safety Net” was introduced to prioritize kidney allocation for patients who have undergone liver transplantation alone (LTA) but still rely on renal replacement therapy or exhibit persistent renal failure within 1 y after liver transplantation.^11,12^

In the context of organ shortage and increasingly restrictive eligibility criteria, it remains unclear whether SLKT confers a survival advantage over LTA, and which patient populations benefit the most. Moreover, the impact of the 2017 OPTN policy on survival outcomes among patients with renal dysfunction of varying severity remains inadequately characterized. To address this, we analyzed the national UNOS/OPTN registry data covering all deceased-donor LTA and SLKT procedures performed between 2010 and 2019. Our study aimed to clarify whether SLKT provides a survival benefit compared with LTA and to assess how the 2017 OPTN policy and “Safety Net” have influenced survival outcomes.

## MATERIALS AND METHODS

### Study Population

This retrospective cohort study was conducted in accordance with the Strengthening the Reporting of Observational Studies in Epidemiology reporting guidelines and used data from UNOS/OPTN deceased-donor registry. Institutional review board approval was waived because the study used de-identified, publicly available registry data in compliance with the UNOS data use agreement.

All adult recipients (≥18 y) deceased-donor liver transplantation performed in the United States between January 1, 2010, and December 31, 2019, were included. Patients with incomplete follow-up data were excluded. The remaining cohort was categorized according to transplant type as LTA or SLKT.

### Renal Function Assessment

Pretransplant and posttransplant renal function were assessed using estimated glomerular filtration rate (eGFR), calculated with the 2021 Chronic Kidney Disease Epidemiology Collaboration (CKD-EPI) equation.^13^ Pretransplant eGFR was derived from the most recent serum creatinine measurement available before transplantation. Patients receiving chronic dialysis before transplantation (defined as dialysis ≥3 mo) were classified in the eGFR <15 mL/min/1.73 m^2^ category to reflect end-stage renal dysfunction.

### Data Collection

Recipient demographics and clinical characteristics were extracted from the UNOS/OPTN registry, including age, sex, body mass index, ethnicity, underlying liver and kidney disease, pretransplant renal function, laboratory MELD score, waiting list time, and transplant year. Donor variables included type (donation after brain death or donation after circulatory death), donor age, and liver/kidney cold ischemia time.

### Study Outcomes

The primary outcome was patient survival. Secondary outcomes included death-censored liver/kidney graft survival and posttransplant eGFR. Death-censored graft survival was defined as graft loss excluding patient death with a functioning graft. Posttransplant eGFR was assessed annually up to 10 y following transplant.

### Statistical Analysis

Continuous variables were reported as medians with interquartile ranges, and categorical variables as numbers with percentages. Baseline characteristics were compared using the Wilcoxon rank-sum test or chi-square test, as appropriate. Kaplan-Meier curves with log-rank tests were used to evaluate patient and graft survival across groups, stratified by pretransplant eGFR. Cox proportional hazards models were used to evaluate the impact of SLKT on patient survival, adjusting for transplant period, recipient age, sex, ethnicity, underlying liver disease, donor age, donor type and cold ischemia time of liver graft as covariates, and tested using Schoenfeld residuals (**Table S1**, **Figure S1**, **SDC**, https://links.lww.com/TP/D405).

To assess the impact of the 2017 OPTN policy change, patients with pretransplant eGFR <45 mL/min/1.73 m^2^, those most affected by revised SLKT eligibility criteria, were stratified into two eras based on transplant date: pre-policy (from January 1, 2010, to August 9, 2017) and post-policy (from August 10, 2017, to December 31, 2019). Given the shorter follow-up in the post-policy cohort, survival analyses focused on 1-, 3, and 5-y outcomes. A *P*-value of <0.05 was considered statistically significant. Data processing, statistical analysis, and data visualization were conducted using R (Version 4.2.2).

## RESULTS

**FIGURE 1.**
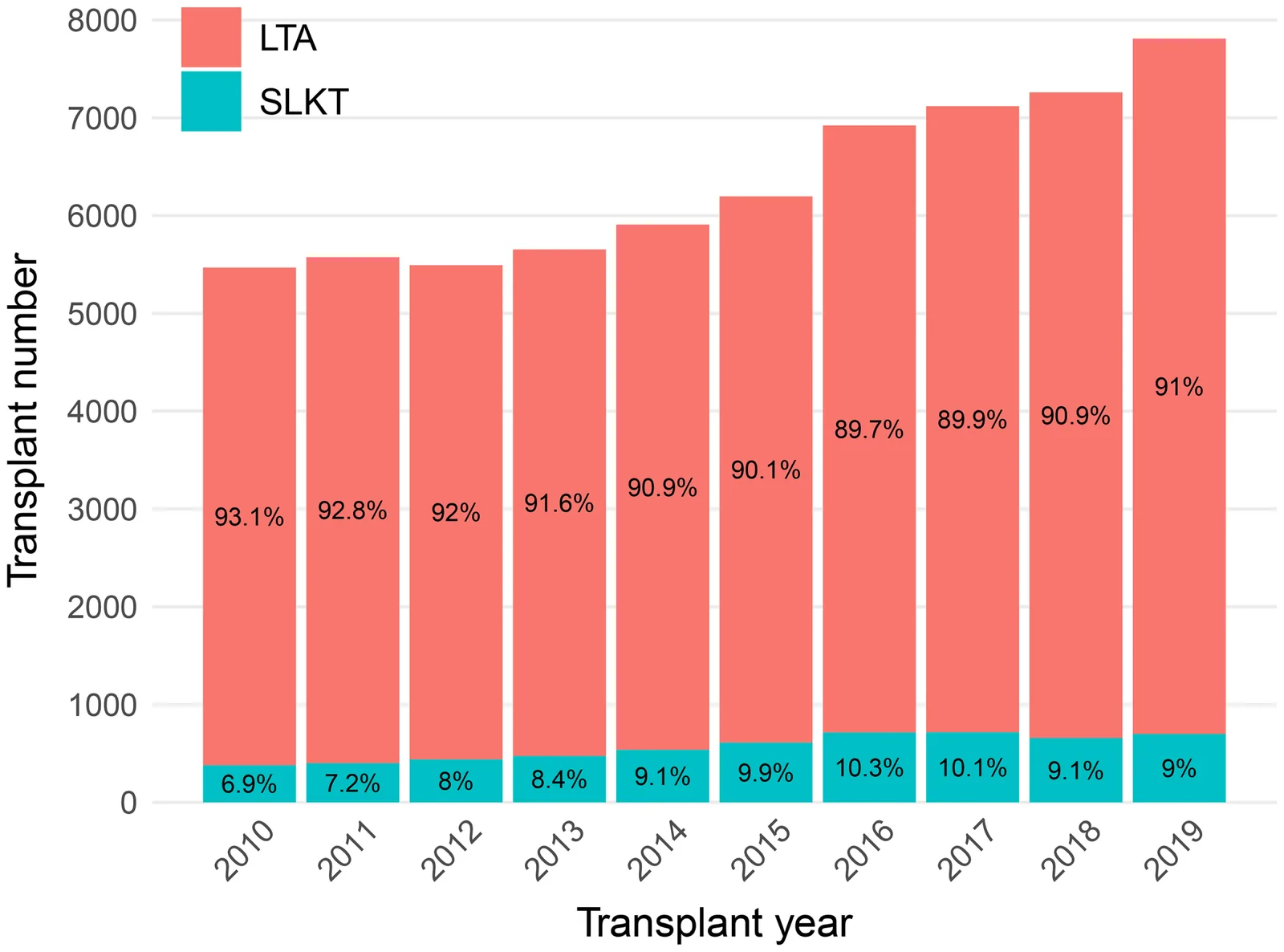
Annual transplant numbers and proportions of LTA and SLKT in the United States, 2010–2019. LTA, liver transplant alone; SLKT, simultaneous liver-kidney transplantation.

Between 2010 and 2019, the UNOS/OPTN registry recorded a total of 63 407 deceased-donor transplants, including 57 780 LTAs and 5627 SLKTs, including 1609 SLKTs after the 2017 OPTN policy implementation (August 10, 2017). Patients were followed up for a median of 5 y (interquartile range, 2.8–7.8 y) after transplantation. The annual number of LTA increased steadily over the past decade. The proportion of SLKT grew from 6.9% of total deceased-donor liver transplants in 2010 to 10.3% in 2016, and dropped to 9% in 2019 after the 2017 OPTN policy restricted SLKT eligibility (Figure 1).

### Baseline Characteristics

Baseline characteristics are summarized in Table 1 and **Table S2** (**SDC**, https://links.lww.com/TP/D405). SLKT recipients were slightly older (median 58 versus 57 y; *P* < 0.001), less frequently male (62.7% versus 66.5%; *P* < 0.001), and had lower body mass index (median 26.7 versus 28.1 kg/m^2^; *P* < 0.001). Ethnic distribution also differed between groups, with SLKT recipients more likely to be Hispanic (17.1% versus 14.1%) or Black (15.1% versus 8.9%), whereas White patients predominated in both groups (62.7% versus 71.0%; *P* < 0.001). As expected, SLKT recipients presented lower pretransplant eGFR (18 versus 68 mL/min/1.73 m^2^; *P* < 0.001) and higher MELD scores (29 versus 21; *P* < 0.001). Donation after circulatory death donors were less common in SLKT than LTA (4.8% versus 6.5%; *P* < 0.001).

**TABLE 1.** Baseline characteristics of LTA and SLKT recipients

	LTA (n = 57 780)	SLKT (n = 5627)	*P*
Age (y)	57 (50–63)	58 (51–63)	<0.001
Sex, male	38 408 (66.5)	3528 (62.7)	<0.001
BMI (kg/m^2^)	28.1 (24.5–32.4)	26.7 (23.3–31.2)	<0.001
Ethnicity			<0.001
White	41 054 (71.0)	3526 (62.7)	
Hispanic	8167 (14.1)	964 (17.1)	
Black	5129 (8.9)	851 (15.1)	
Asian	2526 (4.4)	194 (3.5)	
Others	904 (1.6)	92 (1.6)	
MELD score	21 (13–31)	29 (23–36)	<0.001
Pre-eGFR (mL/min/1.73 m^2^)	68 (42–96)	18 (11–29)	<0.001
Donor			<0.001
DBD	53 997 (93.5)	5355 (95.2)	
DCD	3783 (6.5)	272 (4.8)	
Liver transplant			
Indication			<0.001
Liver cirrhosis	31 262 (54.1)	3892 (69.2)	
Hepatocellular carcinoma	14 977 (25.9)	520 (9.2)	
Cholestatic liver disease	4026 (7.0)	225 (4.0)	
Acute liver failure	2280 (3.9)	83 (1.5)	
Retransplant	1486 (2.6)	179 (3.2)	
Metabolic disease	2279 (3.9)	354 (6.3)	
Polycystic liver disease	209 (0.4)	281 (5.0)	
Others	1261 (2.2)	93 (1.6)	
Waiting list time (d)	89 (16–291)	72 (17–261)	0.004
Cold ischemia time (h)	6.3 (5–8)	6.2 (5–8)	<0.001
Kidney transplant			
Indication			–
Hepatorenal syndrome	–	2526 (44.9)	
Diabetic kidney disease	–	1084 (19.3)	
Glomerulonephritis	–	392 (7.0)	
Interstitial kidney disease	–	355 (6.3)	
Hypertensive kidney disease	–	397 (7.1)	
Polycystic kidney disease	–	310 (5.5)	
Retransplant	–	124 (2.2)	
Metabolic disease	–	98 (1.7)	
Malignancy	–	14 (0.2)	
Others	–	327 (5.8)	
Waiting list time (d)	–	34 (8–150)	–
Cold ischemia time (h)	–	10.3 (8–14)	–

Data are presented as median (interquartile range) or as number (percentage).

BMI, body mass index; DBD, donation after brain death; DCD, donation after circulatory death; LTA, liver transplant alone; MELD, Model for End-stage Liver Disease; pre-eGFR, pretransplant estimated glomerular filtration rate; SLKT, simultaneous liver-kidney transplant.

Table 2 shows that the majority of LTA recipients had preserved renal function before transplantation, with 29.5% having a pretransplant eGFR ≥90 mL/min/1.73 m^2^ and 29% having an eGFR of 60–89 mL/min/1.73 m^2^, while only 3.6% were classified as having a pretransplant eGFR <15 mL/min/1.73 m^2^. In contrast, SLKT recipients predominantly presented with advanced renal dysfunction, with 36.1% having an eGFR 15–29 mL/min/1.73 m^2^ and 38.9% having an eGFR <15 mL/min/1.73 m^2^, while a minority were in the early stages (2% with eGFR ≥90 and 3.7% with eGFR 60–89 mL/min/1.73 m^2^).

**TABLE 2.** Pretransplant eGFR distribution

Pre-eGFR (mL/min/1.73 m^2^)	LTA (n = 57 780)	SLKT^*a*^ (n = 5627)
≥90	17 057 (29.5)	115 (2.0)
60–89	16 746 (29.0)	206 (3.7)
45–59	8308 (14.4)	301 (5.3)
30–44	7444 (12.9)	771 (13.7)
15–29	6157 (10.6)	2034 (36.1)
<15	2068 (3.6)	2187 (38.9)

a 13 (0.2%) missing data.

LTA, liver transplant alone; pre-eGFR, pretransplant estimated glomerular filtration rate; SLKT, simultaneous liver-kidney transplant.

### Patient Survival

**FIGURE 2.**
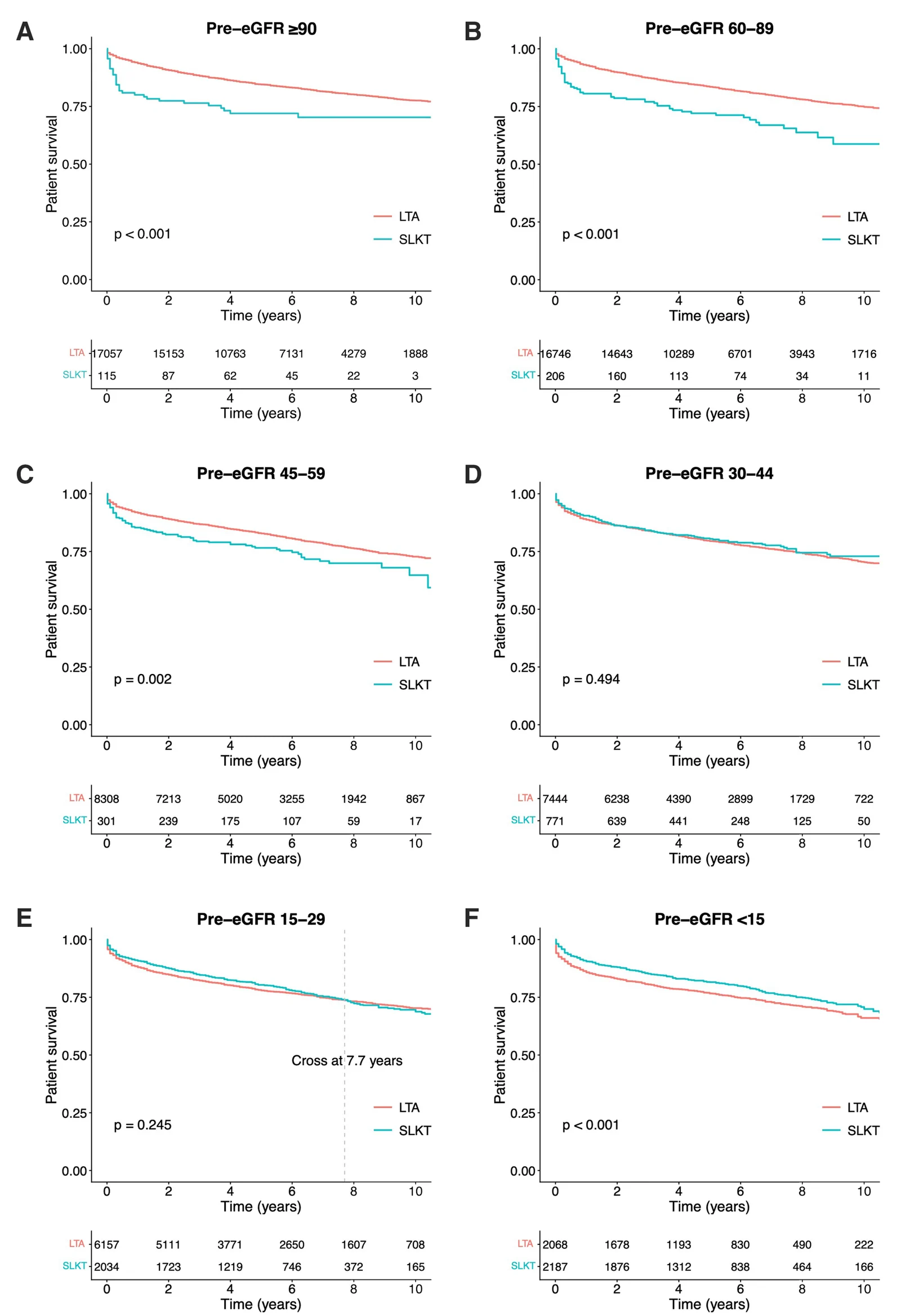
Patient survival after LTA and SLKT stratified by pretransplant eGFR. A, Pretransplant eGFR ≥90 mL/min/1.73 m^2^. B, Pretransplant eGFR 60–89 mL/min/1.73 m^2^. C, Pretransplant eGFR 45–59 mL/min/1.73 m^2^. D, Pretransplant eGFR 30–44 mL/min/1.73 m^2^. E, Pretransplant eGFR 15–29 mL/min/1.73 m^2^. F, Pretransplant eGFR <15 mL/min/1.73 m^2^. eGFR, estimated glomerular filtration rate; LTA, liver transplant alone; SLKT, simultaneous liver-kidney transplantation.

Patient survival stratified by pretransplant eGFR is shown in Figure 2. Among patients with preserved or mildly impaired renal function (eGFR ≥90, 60–89, and 45–59 mL/min/1.73 m^2^), posttransplant survival following LTA was significantly higher than SLKT (*P* < 0.001, *P* < 0.001, and *P* = 0.002, respectively; Figure 2A–C). In patients with pretransplant eGFR 30–44 mL/min/1.73 m^2^, survival curves were similar (1-, 3-, 5-, and 10-y survival: 88.8%, 83.9%, 79.5%, and 70.4% for LTA versus 90.5%, 84.2%, 80.5%, and 72.9% for SLKT; *P* = 0.494; Figure 2D). In contrast, among patients with pretransplant eGFR 15–29 mL/min/1.73 m^2^, survival curves favored SLKT during early follow-up but converged over time, with crossing observed at approximately 7.7 y posttransplant. One-, 3-, 5-, and 10-y survival was 88%, 82.2%, 78%, and 70.3% for LTA compared with 90.8%, 84.6%, 80.4%, and 68.8% for SLKT (*P* = 0.245; Figure 2E). For patients with pretransplant eGFR <15 mL/min/1.73 m^2^, SLKT provided significantly improved survival compared with LTA (1-, 3-, 5-, and 10-y survival: 85.8%, 80.6%, 76.6%, and 66% for LTA versus 90.5%, 85.2%, 81.6%, and 69.9% for SLKT; *P* < 0.001; Figure 2F).

### Death-censored Graft Survival

Death-censored liver survival is presented in **Figure S2** (**SDC**, https://links.lww.com/TP/D405). For patients with pretransplant eGFR ≥90, 60–89, 45–59, and 30–44 mL/min/1.73 m^2^, liver survival did not differ significantly between LTA and SLKT (*P* = 0.196, *P* = 0.821, *P* = 0.335, and *P* = 0.423, respectively; **Figure S2A–D** [**SDC**, https://links.lww.com/TP/D405]). In contrast, SLKT was associated with significantly improved liver survival in patients with pretransplant eGFR 15–29 mL/min/1.73 m^2^, 1-, 3-, 5-, and 10-y graft survival was 95.7%, 94.1%, 93%, and 92% for LTA versus 97.3%, 96.3%, 95.6%, and 94.1% for SLKT (*P* < 0.001; **Figure S2E**, **SDC**, https://links.lww.com/TP/D405). Similarly, in patients with pretransplant eGFR <15 mL/min/1.73 m^2^, liver survival was significantly higher after SLKT than LTA. One-, 3-, 5-, and 10-y survival was 95.5%, 94.6%, 93.7%, and 91.1% for LTA versus 97.2%, 96.1%, 95.3%, and 92.7% for SLKT (*P* = 0.028; **Figure S2F**, **SDC**, https://links.lww.com/TP/D405).

Death-censored kidney survival was excellent overall in SLKT recipients, although modest differences were observed across pretransplant eGFR strata (*P* = 0.049; **Figure S3**, **SDC**, https://links.lww.com/TP/D405). At 1, 3, 5, and 10 y posttransplant, kidney survival ranged from 93.8% to 96.7%, 91.2% to 95.1%, 88% to 92.9%, and 83.8% to 88.8%, respectively, across all pretransplant eGFR categories.

### Posttransplant Renal Function

Among patients remaining under follow-up at each time point, completeness of posttransplant renal function data were 95.5% at 6 mo and 93.4% at 1 y, gradually declining to 76.7% at 5 y and 43.7% at 10 y (**Table S3**, **SDC**, https://links.lww.com/TP/D405).

Posttransplant renal function is shown in **Figure S4** (**SDC**, https://links.lww.com/TP/D405). Posttransplant renal function differed between groups across baseline eGFR strata. Among patients with baseline eGFR ≥90 mL/min/1.73 m^2^, eGFR values were slightly higher in the LTA group throughout follow-up. In contrast, in patients with pretransplant eGFR <60 mL/min/1.73 m^2^, SLKT recipients demonstrated consistently higher median eGFR values compared with LTA recipients during long-term follow-up, with median differences of 7–12 mL/min/1.73 m^2^. The most pronounced separation was observed in patients with severely impaired renal function (eGFR <30 mL/min/1.73 m^2^).

### Stage- and Time-dependent Survival Impact of SLKT Versus LTA

We assessed the independent effect of SLKT versus LTA with cumulative 1-, 3-, 5-, and 10-y patient survival among patients with pretransplant eGFR <60 mL/min/1.73 m^2^ using multivariable Cox regression models.

**FIGURE 3.**
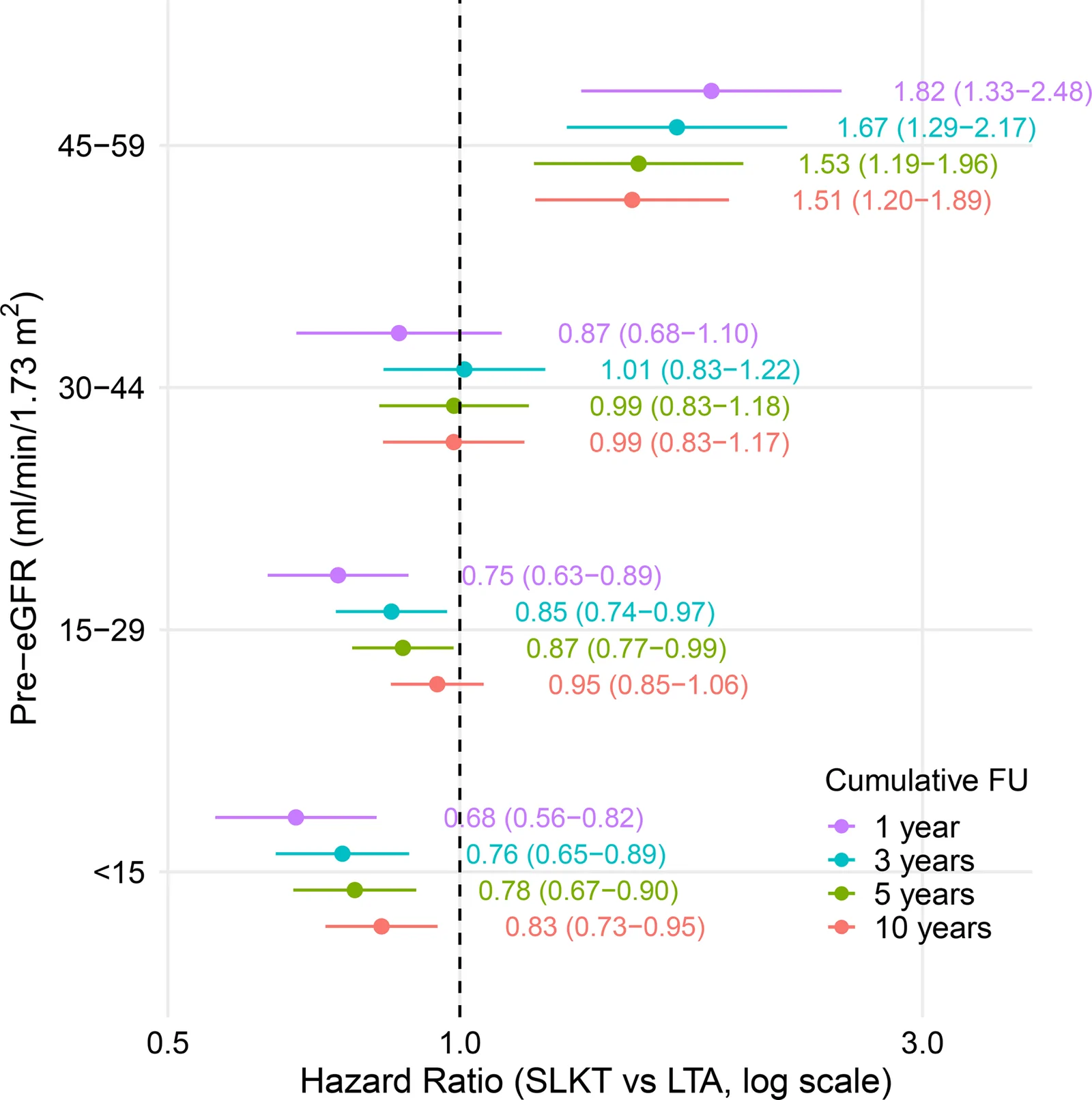
Multivariable Cox regression forest plot comparing patient survival after SLKT vs LTA stratified by pretransplant eGFR. Hazard ratios are adjusted for transplant period, recipient age, sex, ethnicity, underlying liver disease, donor age, donor type, and liver graft cold ischemia time. eGFR, estimated glomerular filtration rate; FU, follow-up; LTA, liver transplant alone; SLKT, simultaneous liver-kidney transplantation.

As shown in Figure 3, for patients with pretransplant eGFR 45–59 mL/min/1.73 m^2^, SLKT was consistently associated with a significantly higher risk of mortality compared with LTA. Adjusted hazard ratios (HRs) for cumulative mortality were 1.82 (95% confidence interval [CI], 1.33-2.48; *P* < 0.001) at 1 y, 1.67 (95% CI, 1.29-2.17; *P* < 0.001) at 3 y, 1.53 (95% CI, 1.19-1.96; *P* < 0.001) at 5 y, and 1.51 (95% CI, 1.20-1.89; *P* < 0.001) at 10 y. In patients with pretransplant eGFR 30–44 mL/min/1.73 m^2^, no significant differences in cumulative mortality were observed between SLKT and LTA at 1, 3, 5, or 10 y (*P* = 0.244, *P* = 0.911, *P* = 0.864, and *P* = 0.837, respectively). In contrast, for patients with pretransplant eGFR 15–29 mL/min/1.73 m^2^, SLKT reduced cumulative mortality by 25% at 1 y (HR, 0.75; 95% CI, 0.63-0.89; *P* < 0.001), 15% at 3 y (HR, 0.85; 95% CI, 0.74-0.97; *P* = 0.017), and 13% at 5 y (HR, 0.87; 95% CI, 0.77-0.99; *P* = 0.028), respectively, whereas no significant survival difference was observed at 10 y (*P* = 0.333). For patients with pretransplant eGFR <15 mL/min/1.73 m^2^, SLKT conferred a consistent survival benefit throughout the 10-y follow-up period. Compared with LTA, cumulative mortality was reduced by 32% at 1 y (HR, 0.68; 95% CI, 0.56-0.82; *P* < 0.001), 24% at 3 y (HR, 0.76; 95% CI, 0.65-0.89; *P* < 0.001), 22% at 5 y (HR, 0.78; 95% CI, 0.67-0.90; *P* < 0.001), and 17% at 10 y (HR, 0.83; 95% CI, 0.73-0.95; *P* = 0.004).

### Impact of the 2017 OPTN Policy on Transplant Practice and Survival

**FIGURE 4.**
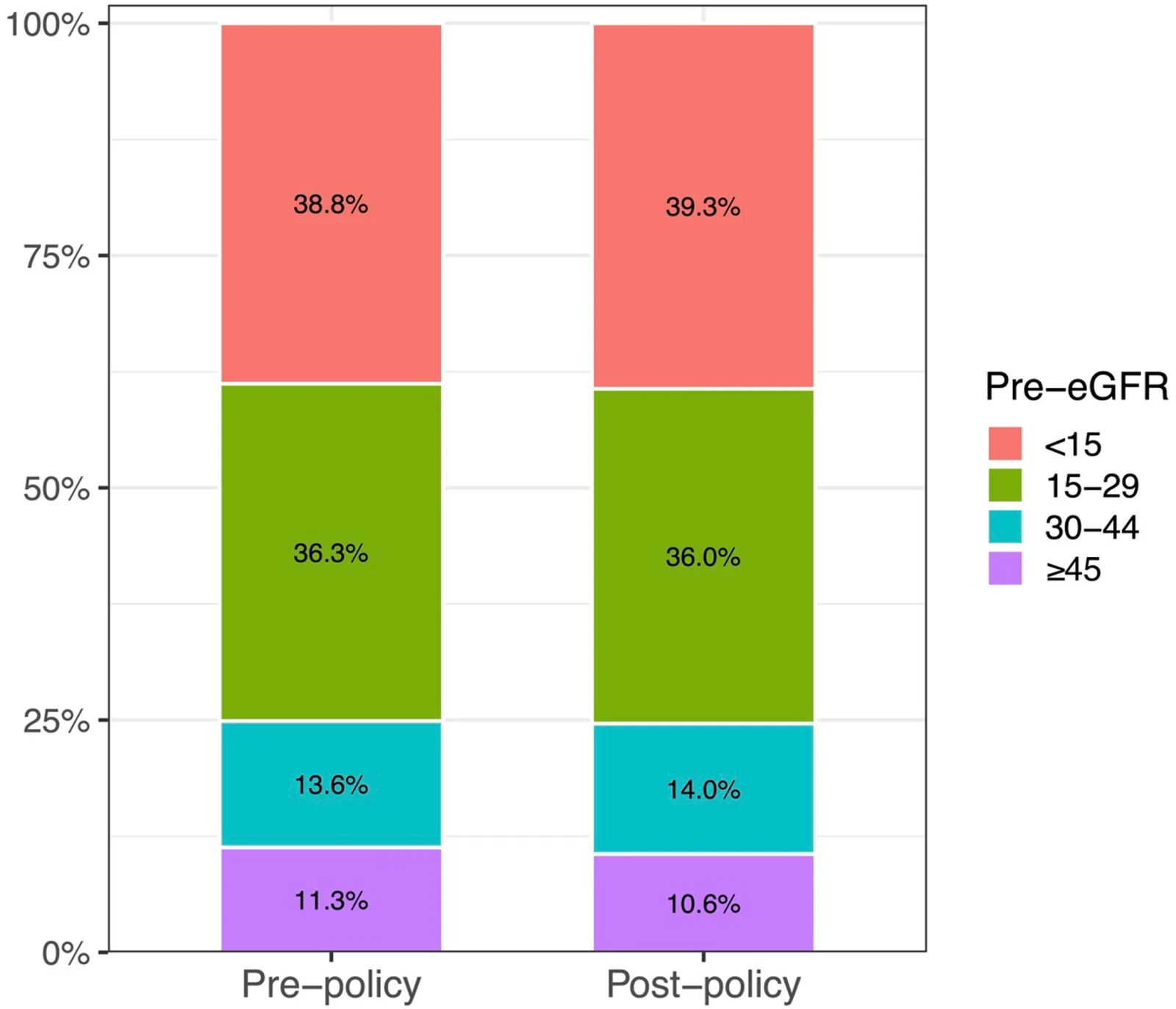
Distribution of simultaneous liver-kidney transplantation (SLKT) stratified by pretransplant eGFR before and after implementation of the 2017 Organ Procurement and Transplant Network (OPTN) policy. eGFR, estimated glomerular filtration rate.

As shown in Figure 4, implementation of the 2017 OPTN policy was associated with modest shifts in SLKT procedures across pretransplant eGFR strata. Compared with the pre-policy era, the proportion of SLKT recipients with preserved or mildly impaired renal function (pretransplant eGFR ≥45 mL/min/1.73 m^2^) decreased by 0.7% in the post-policy era. In contrast, the proportion of SLKT recipients with pretransplant eGFR <15 mL/min/1.73 m^2^ increased by 0.5%. Smaller shifts were observed in intermediate strata, with a 0.4% increase among patients with eGFR 30–44 mL/min/1.73 m^2^ and a 0.3% decrease among those with eGFR 15–29 mL/min/1.73 m^2^.

**FIGURE 5.**
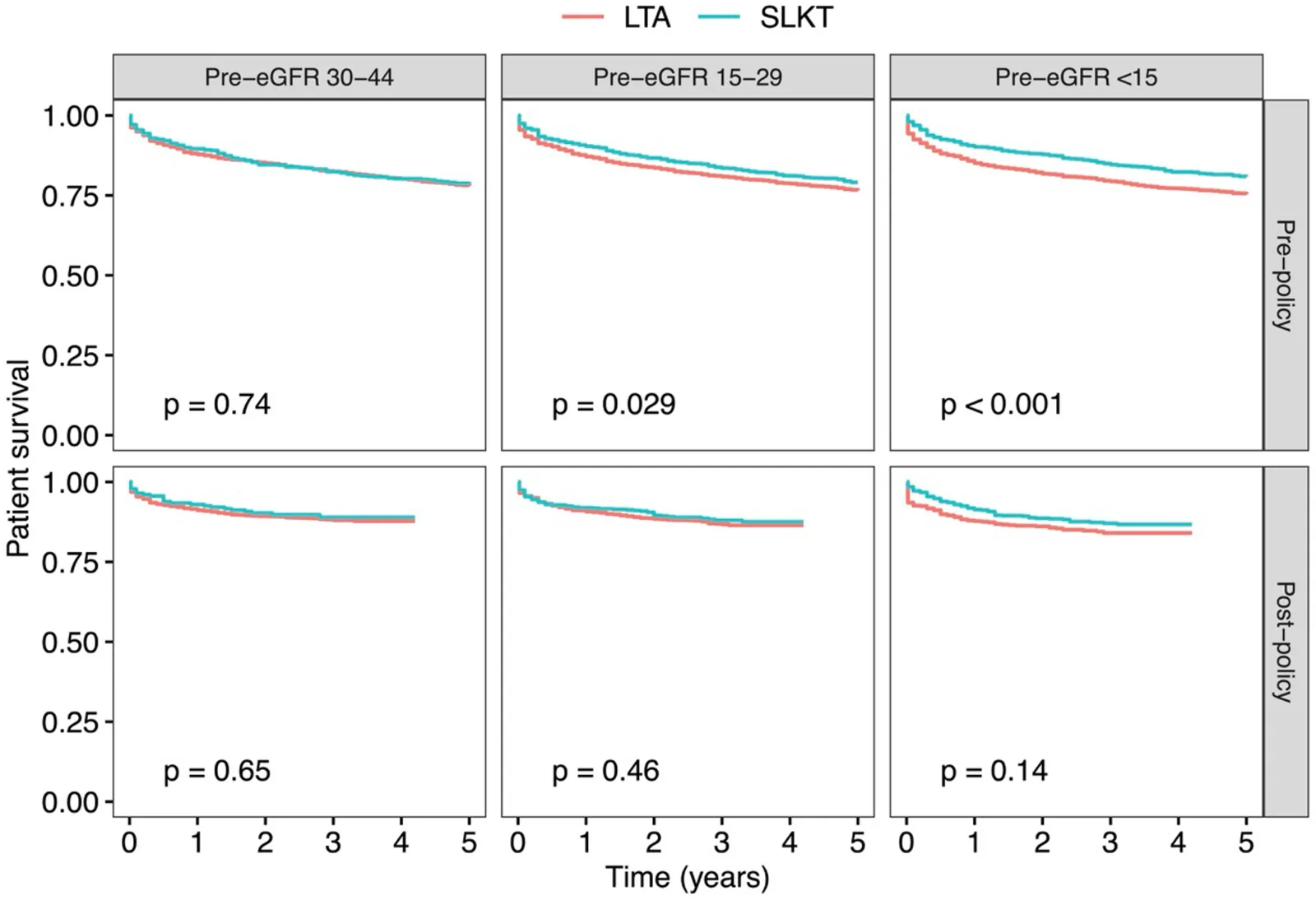
Comparison of patient survival after LTA vs SLKT before and after implementation of the 2017 Organ Procurement and Transplant Network (OPTN) policy. eGFR, estimated glomerular filtration rate; LTA, liver transplant alone; SLKT, simultaneous liver-kidney transplantation.

We further assessed the impact of OPTN policy change on patient survival among recipients with pretransplant eGFR <45 mL/min/1.73 m^2^, the population most affected by the more stringent SLKT eligibility criteria (Figure 5). Patient survival following LTA and SLKT was analyzed separately for the pre-policy and post-policy eras. Among patients transplanted with pretransplant eGFR 30–44 mL/min/1.73 m^2^, no significant survival differences were observed between SLKT and LTA in either the pre-policy or post-policy periods (*P* = 0.74 and *P* = 0.65, respectively). In contrast, in the pre-policy era, SLKT was associated with a significant survival advantage among patients with more advanced renal dysfunction. Specifically, for patients with pretransplant eGFR 15–29 mL/min/1.73 m^2^, 1-, 3-, and 5-y survival was 90.3%, 83.5%, and 79.1% after SLKT compared with 87.1%, 80.9%, and 76.5% after LTA (*P* = 0.029). In patients with pretransplant eGFR <15 mL/min/1.73 m^2^, 1-, 3-, and 5-y survival was 90.2%, 84.6%, and 80.8% after SLKT versus 85.1%, 79.4%, and 75.3% after LTA (*P* < 0.001). However, after the revised OPTN policy implementation, these survival differences were no longer significant. In the post-policy era, patient survival did not differ significantly between SLKT and LTA among recipients with pretransplant eGFR 15–29 mL/min/1.73 m^2^ or <15 mL/min/1.73 m^2^ during the available follow-up period (*P* = 0.46 and *P* = 0.14, respectively).

Notably, in the post-policy era, 221 kidney after liver transplantation (KALT) procedures (1.4% of all LTAs) were recorded under the “Safety Net” allocation priority within 1-y post-LTA.

## DISCUSSION

Using the OPTN/UNOS national registry, we systematically compared outcomes of SLKT and LTA across strata of pretransplant renal function. Our findings demonstrate a clear stage- and time-dependent survival effect of SLKT. Among patients with pretransplant eGFR ≥30 mL/min/1.73 m^2^, SLKT did not confer a survival advantage over LTA; instead, recipients undergoing LTA achieved comparable or better long-term survival. In contrast, SLKT was associated with improved survival among patients with advanced renal dysfunction (pretransplant eGFR <30 mL/min/1.73 m^2^).

These findings extend prior evidence regarding the comparative effectiveness of SLKT and LTA in patients with renal dysfunction. In our previous systematic review and meta-analysis, SLKT recipients demonstrated improved liver graft survival compared with LTA recipients, despite comparable overall patient survival, suggesting potential benefit in selected populations.^14^ However, limited studies have been able to identify patients of which extents of renal dysfunction can benefit from SLKT. Analyses from the UK national transplant registry reported that only patients dependent on renal replacement therapy experienced survival benefits from SLKT, with 5-y survival of 90.6% versus 71% following LTA.^15^ Similarly, data from the Dutch Organ Transplantation Registry demonstrated improved survival with SLKT in patients with CKD stage 5 or receiving dialysis, with 5-y survival of 80.8% compared with 51.7% after LTA.^16^ Our study refines these findings by demonstrating the stage-specific and time-dependent impact of SLKT, which provides a more granular basis for patient selection and may better align organ allocation with clinical benefit.

The 2017 OPTN policy restricted SLKT eligibility to candidates with sustained eGFR ≤60 mL/min/1.73 m^2^ for >90 d, and most recent GFR ≤30 mL/min/1.73 m^2^ or ongoing dialysis.^17^ Meanwhile, the “Safety Net” policy ensures that LTA recipients who develop persistent renal failure have priority for subsequent KALT.^18,19^ These complementary strategies are intended to balance appropriate dual-organ allocation and optimize kidney utilization given the prolonged deceased-donor kidney waiting times (typically >3 y).^20^ Altshuler et al^19^ found that after implementing “Safety Net” policies, the proportion of SLKT cases among total liver transplant recipients decreased from 10.2% to 9.0%, while the number of KALT procedures increased by 65% >2.5 y. Cheng et al^21^ demonstrated that such policies improved organ utilization and liver transplant outcomes. International experience supports these findings. For example, in the Eurotransplant region, patients who require dialysis after the other organ transplant between 90 and 360 d will receive bonus points for a delayed kidney transplantation.^22,23^

Consistent with the intent of this policy framework, both the number and proportion of SLKT procedures declined following implementation of the revised eligibility criteria. The survival advantage of SLKT observed among patients with pretransplant eGFR <30 mL/min/1.73 m^2^ in the pre-policy era was no longer evident in the post-policy era. Under the more stringent SLKT policy, the proportion of LTA recipients increased by approximately 1%, suggesting that a subset of patients with concurrent renal dysfunction who would previously have undergone SLKT instead received LTA. Despite inclusion of this potentially higher-risk population, post-policy survival remained comparable between LTA and SLKT recipients. This finding reflects recovery of reversible acute kidney injury after LTA, and timely KALT through the “Safety Net” priority allocation, which contributed to 1.4% LTA recipients within 1 y after liver transplantation. Such findings are in line with the study by Cannon et al^24^ demonstrating that LTA patients with renal dysfunction who are protected by “Safety Net” achieve comparable 1-y survival to those not requiring kidney transplantation, and superior outcomes compared with SLKT listed patients undergoing LTA without subsequent KALT listing. Additionally, survival improved in both SLKT and LTA recipients during the post-policy era compared with the earlier period, likely reflecting broader advances in surgical technique, perioperative care, and posttransplant management rather than policy effects alone.

Several limitations must be addressed. First, pretransplant renal function may have been overestimated in a subset of patients. In patients with advanced cirrhosis, reduced muscle mass and lower serum creatinine levels can lead to overestimation of true renal function when using creatinine-based CKD-EPI equations.^25^ Prior studies have demonstrated that among liver disease patients with measured GFR <30 mL/min/1.73 m^2^, CKD-EPI may overestimate true GFR by approximately 8.7 mL/min/1.73 m^2^.^26^ Accordingly, some patients categorized in this study as having pretransplant eGFR 30–44 mL/min/1.73 m^2^ may have had true GFR <30 mL/min/1.73 m^2^. In addition, the UNOS dataset does not capture acute dialysis at the time of transplantation. As a result, patients who underwent urgent pretransplant dialysis but subsequently had relatively low recorded serum creatinine may have been misclassified into higher eGFR strata (eg, ≥60 mL/min/1.73 m^2^), which may explain the presence of SLKT in patients with apparently preserved renal function. These factors may have reduced the accuracy of using a single eGFR value for renal function stratification. Importantly, because creatinine-based estimation in cirrhosis generally results in overestimation rather than underestimation of renal dysfunction, misclassification would most likely shift patients with more severe renal impairment into higher eGFR categories. Therefore, the observed survival benefit among patients categorized as eGFR <30 mL/min/1.73 m^2^ is unlikely to be inflated and represents a conservative estimate. Nevertheless, future studies incorporating more accurate assessment of renal function in patients with liver disease, such as cystatin C-based equations or the GRAIL (Glomerular Filtration Rate Assessment in Liver Disease) model, are warranted to validate these findings.^26^

In conclusion, SLKT provides a survival benefit in patients with pretransplant eGFR <30 mL/min/1.73 m^2^; however, this difference was no longer significant following implementation of the 2017 OPTN policy and “Safety Net” framework. Importantly, the revised policy was associated with reduced SLKT procedures without compromising patient survival, as post-policy LTA recipients achieved comparable outcomes to SLKT recipients. These findings highlight the critical role of pretransplant renal function in guiding organ allocation, supporting SLKT for patients with severe and irreversible renal dysfunction while favoring LTA for those with preserved or moderately impaired renal function, thereby optimizing organ utilization without sacrificing long-term patient survival.

## Supplementary Material


