# Supplementary material for: Simultaneous Liver-kidney Versus Liver Transplantation Categorized by Pretransplant eGFR: Impact of the 2017 OPTN Policy
Source: Transplantation. 2026 Jun 30;110(10):e2096–104. doi: 10.1097/TP.0000000000005776 (PMC13585157; doi:10.1097/TP.0000000000005776)
Supplement: Supplementary file 1 [file tpa-110-e2096-s001.pdf]

Supplementary

Table S1. Univariable Cox regression analysis of recipient, donor, and transplant characteristics associated with posttransplant survival

	HR (95% CI)	p value
LTA	ref	
SLKT	0.997 (0.935-1.063)	0.922
Transplant period		
2010-2012	ref	
2013-2015	0.782 (0.737-0.830)	<0.001
2016-2019	0.600 (0.563-0.639)	<0.001
Age (year)	1.017 (1.015-1.020)	<0.001
Sex		
Female	ref	
Male	1.115 (1.060-1.173)	<0.001
Ethnicity		
White	ref	
Hispanic	0.867 (0.804-0.934)	<0.001
Black	1.214 (1.122-1.314)	<0.001
Asian	0.930 (0.805-1.075)	0.326
Others	1.002 (0.828-1.211)	0.986
Liver transplant indication		
Liver cirrhosis	ref	
Hepatocellular carcinoma	1.292 (1.208-1.382)	<0.001
Cholestatic liver disease	0.768 (0.683-0.863)	<0.001
Acute liver failure	0.896 (0.785-1.022)	0.102
Retransplant	0.716 (0.605-0.847)	<0.001
Metabolic disease	1.664 (1.519-1.822)	<0.001
Polycystic liver disease	0.451 (0.325-0.626)	<0.001
Others	1.395 (1.164-1.673)	<0.001
MELD score		
<15	ref	
15-25	0.937 (0.846-1.038)	0.215
>25	0.954 (0.866-1.051)	0.342
Pre-eGFR (ml/min/1.73m^2)		
45-59	ref	
30-44	1.143 (1.070-1.221)	<0.001
15-29	1.196 (1.120-1.276)	<0.001
<15	1.212 (1.121-1.310)	<0.001
Donor age (year)	1.007 (1.005-1.008)	<0.001
Donor type		
DBD	ref	
DCD	1.117 (1.003-1.243)	0.043
Cold ischemia time (hour)	1.019 (1.010-1.028)	<0.001

DBD, donation after brain death; DCD, donation after circulatory death; LTA, liver transplant alone; MELD, Model for End-stage Liver Disease; Pre-eGFR, pretransplant estimated glomerular filtration rate; SLKT, simultaneous liver kidney transplant.

Table S2. Kidney transplant indication categorized by pretransplant eGFR

Pre-eGFR (ml/min/1.73 m^2)	≥90 (n=146)	60-89 (n=261)	45-59 (n=429)	30-44 (n=1114)	15-29 (n=3035)	<15 (n=3371)
Hepatorenal syndrome	86 (58.9)	145 (55.6)	231 (53.8)	502 (45.1)	1305 (43)	1078 (32)
Diabetic kidney disease	15 (10.3)	29 (11.1)	61 (14.2)	197 (17.7)	566 (18.6)	684 (20.3)
Glomerulonephritis	4 (2.7)	13 (5.0)	27 (6.3)	94 (8.4)	250 (8.2)	333 (9.9)
Interstitial kidney disease	13 (8.9)	31 (11.9)	30 (7.0)	98 (8.8)	273 (9.0)	238 (7.1)
Hypertensive kidney disease	6 (4.1)	10 (3.8)	22 (5.1)	63 (5.7)	217 (7.1)	304 (9.0)
Polycystic kidney disease	2 (1.4)	4 (1.5)	10 (2.3)	34 (3.1)	99 (3.3)	283 (8.4)
Retransplant	5 (3.4)	5 (1.9)	12 (2.8)	22 (2.0)	85 (2.8)	152 (4.5)
Metabolic disease	2 (1.4)	-	3 (0.7)	6 (0.5)	34 (1.1)	99 (2.9)
Malignancy	1 (0.7)	1 (0.4)	1 (0.2)	5 (0.4)	5 (0.2)	6 (0.2)
Others	12 (8.2)	23 (8.8)	32 (7.5)	93 (8.3)	201 (6.6)	194 (5.8)

Pre-eGFR, pretransplant estimated glomerular filtration rate.

Table S3. Risk-set-adjusted completeness of posttransplant eGFR measurements

Follow-up time	Patients at risk	eGFR available	Completeness (%)
6 months	59615	56937	95.5
1 year	58182	54345	93.4
2 years	54773	46392	84.7
3 years	46213	38228	82.7
4 years	38760	30965	79.9
5 years	31765	24371	76.7
6 years	25535	18933	74.1
7 years	19926	14029	70.4
8 years	15075	9837	65.3
9 years	10636	6103	57.4
10 years	6541	2860	43.7

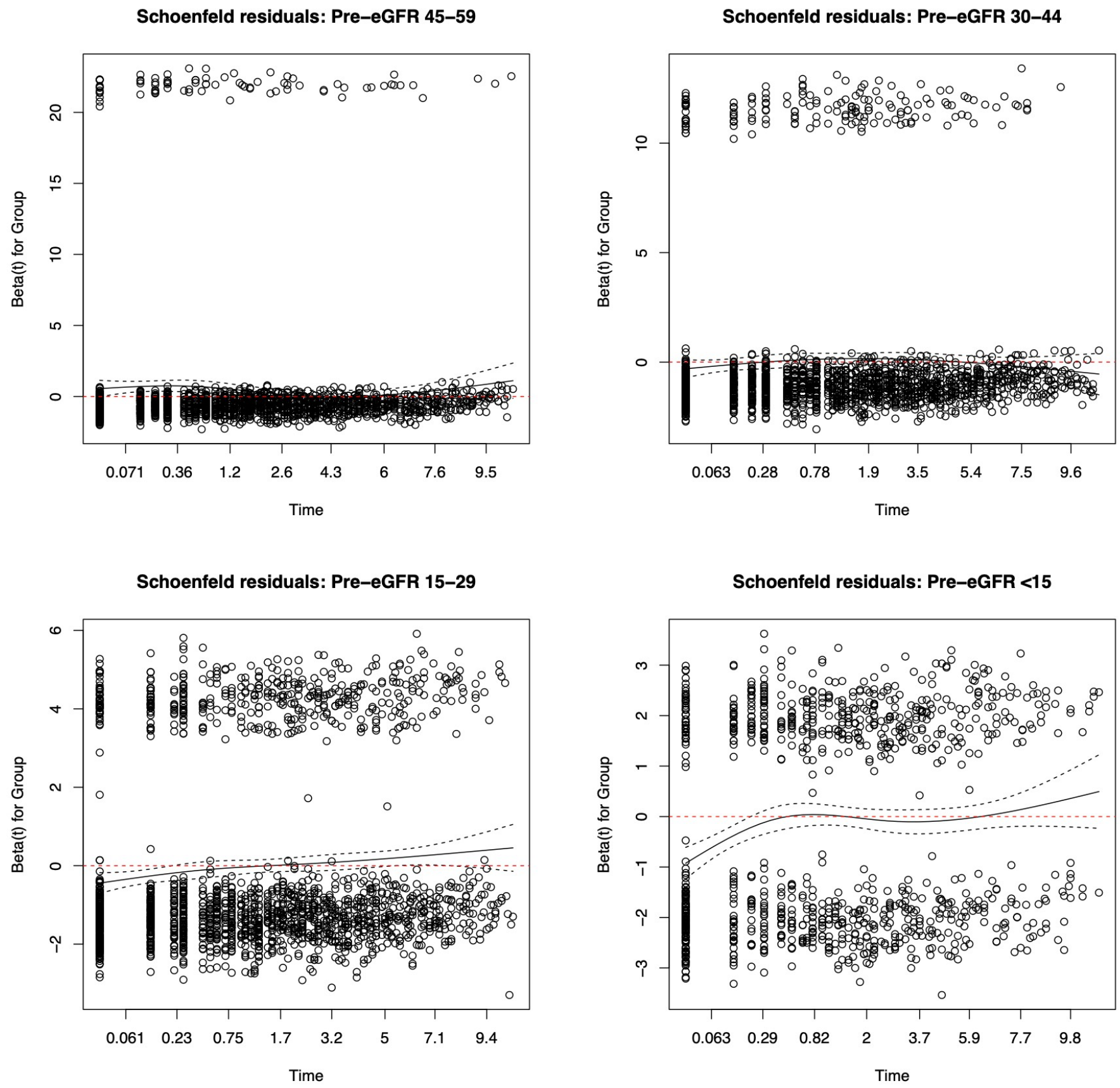

Figure S1. Schoenfeld residual plots for SLKT versus LTA stratified by pretransplant eGFR. The solid line represents a smoothed fit of the residuals over time (with confidence intervals), and the dashed red line indicates zero. A stable, near-horizontal trend around zero suggests that the proportional hazards assumption holds for the variable (SLKT versus LTA) in each pretransplant eGFR subgroup.

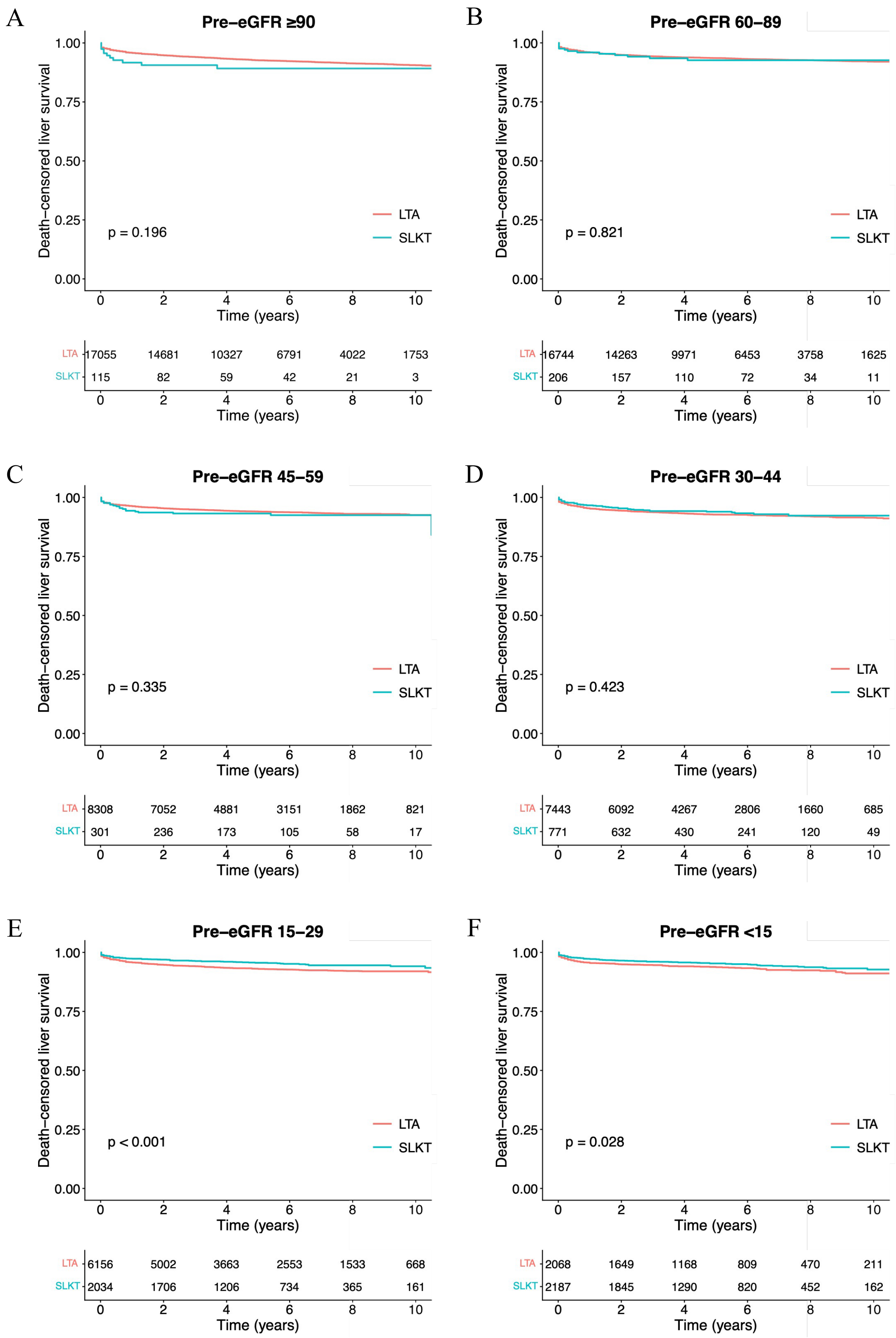

Figure S2. Death-censored liver graft survival after liver transplant alone (LTA) and simultaneous liver-kidney transplantation (SLKT) stratified by pretransplant eGFR.

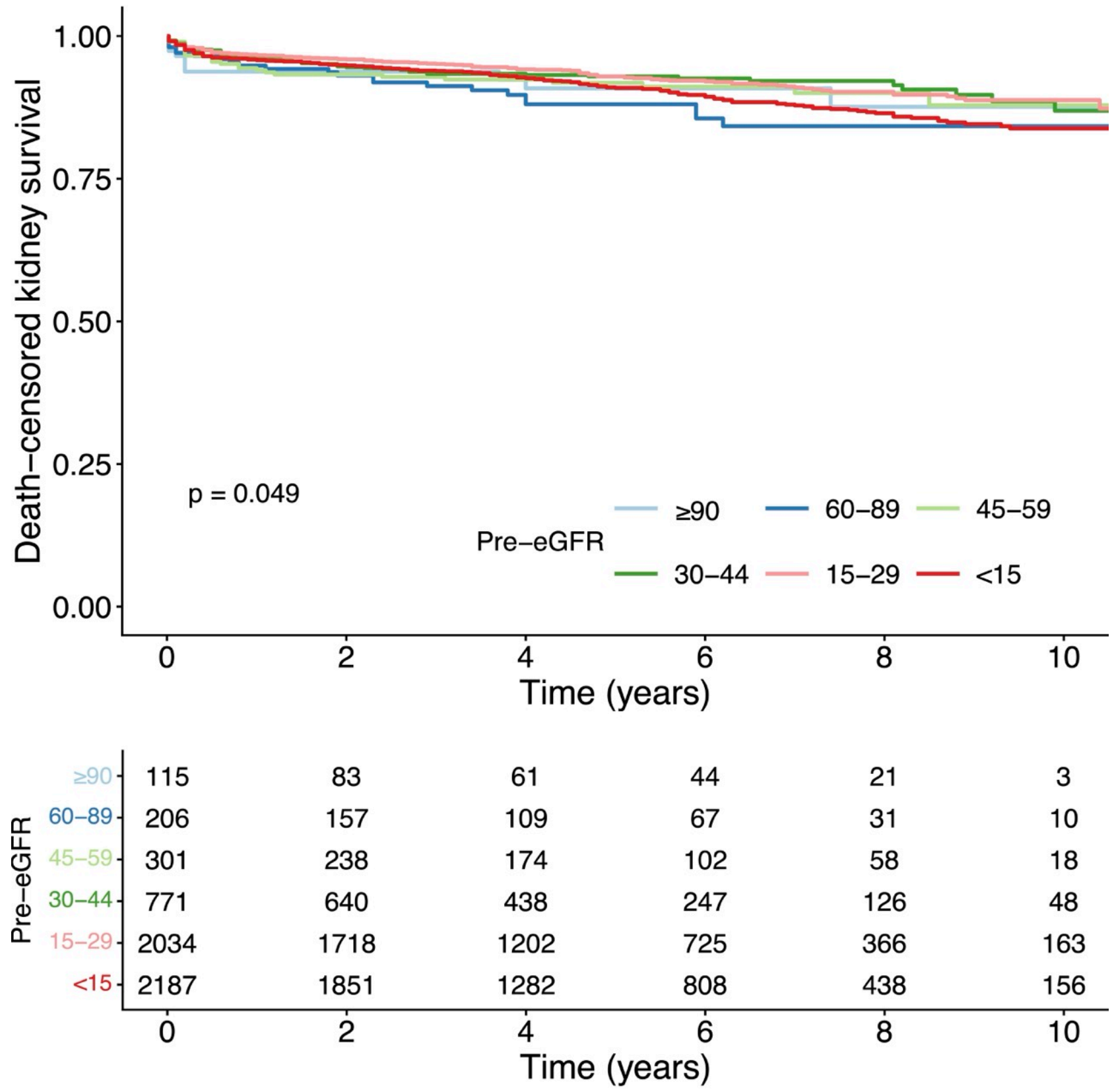

Figure S3. Death-censored kidney graft survival after simultaneous liver-kidney transplantation (SLKT) stratified by pretransplant eGFR.

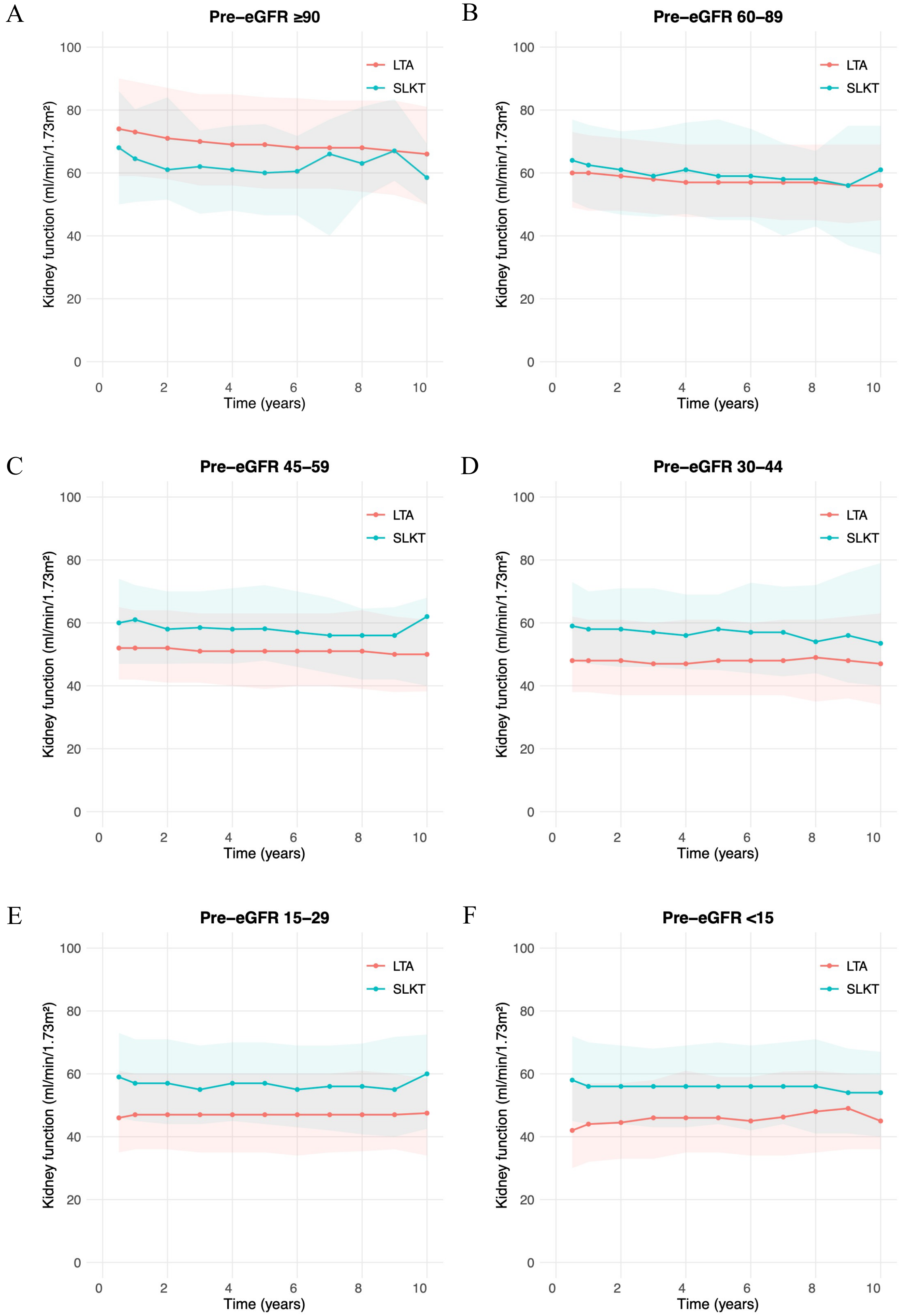

Figure S4. Posttransplant eGFR in LTA and SLKT recipients stratified by pretransplant eGFR.
